# Neurophysiological Correlates of Frequency, Concreteness, and Iconicity in American Sign Language

**DOI:** 10.1162/nol_a_00012

**Published:** 2020-07-07

**Authors:** Karen Emmorey, Kurt Winsler, Katherine J. Midgley, Jonathan Grainger, Phillip J. Holcomb

**Affiliations:** 1 School of Speech, Language and Hearing Sciences, San Diego State University; 2 Department of Psychology, University of California, Davis; 3 Department of Psychology, San Diego State University; 4 Laboratoire de Psychologie Cognitive, Aix-Marseille University, Centre National de la Recherche Scientifique

**Keywords:** American Sign Language, event-related potentials, frequency, concreteness, iconicity, lexical access

## Abstract

To investigate possible universal and modality-specific factors that influence the neurophysiological response during lexical processing, we recorded event-related potentials while a large group of deaf adults (*n* = 40) viewed 404 signs in American Sign Language (ASL) that varied in ASL frequency, concreteness, and iconicity. Participants performed a go/no-go semantic categorization task (does the sign refer to people?) to videoclips of ASL signs (clips began with the signer’s hands at rest). Linear mixed-effects regression models were fit with per-participant, per-trial, and per-electrode data, allowing us to identify unique effects of each lexical variable. We observed an early effect of frequency (greater negativity for less frequent signs) beginning at 400 ms postvideo onset at anterior sites, which we interpreted as reflecting form-based lexical processing. This effect was followed by a more widely distributed posterior response that we interpreted as reflecting lexical-semantic processing. Paralleling spoken language, more concrete signs elicited greater negativities, beginning 600 ms postvideo onset with a wide scalp distribution. Finally, there were no effects of iconicity (except for a weak effect in the latest epochs; 1,000–1,200 ms), suggesting that iconicity does not modulate the neural response during sign recognition. Despite the perceptual and sensorimotoric differences between signed and spoken languages, the overall results indicate very similar neurophysiological processes underlie lexical access for both signs and words.

## INTRODUCTION

Current theories in linguistics, psychology, and cognitive neuroscience have all been developed primarily from investigations of spoken languages. This focus has led theories to ignore or downplay phenomena that are limited in speech but are pervasive in sign languages, such as iconicity (a nonarbitrary relation between phonological form and meaning) and observable linguistic articulators (the vocal articulators for speech are largely hidden from view). By widening our scientific lens to include sign languages, we can distinguish neurobiological principles that are universal to human language processing from those that are modulated by the specific sensorimotor systems within which language is instantiated. To investigate possible universal factors in language processing, the present study used event-related potentials (ERPs) to determine the impact of lexical frequency and concreteness on the brain’s response to a large set of signs (*n* = ~400) from American Sign Language (ASL) in a large group of deaf signers (*n* = 40). The frequency and semantic properties of lexical forms are likely to be represented and processed similarly for signed and spoken languages, although the time-course and scalp distribution of these effects could differ due to differences between the visual-manual and auditory-vocal modalities. In addition, we investigated whether iconicity, a phenomenon influenced by the modality of sign language, affects the time course or amplitude of neural responses when signers comprehend ASL signs.

One challenge to investigating the effects of frequency on language processing is that currently there are no ASL corpora available from which frequency counts can be obtained. Psycholinguistic research has thus relied on sign familiarity ratings by deaf signers to estimate lexical frequency (e.g., Carreiras, Guitiérrez-Sigut, Baquero, & Corina, 2008; Emmorey, 1991; Emmorey, Petrich, & Gollan, 2013). Recently, a database of ~1,000 ASL signs (ASL-LEX) was created that contains frequency ratings from 25 to 30 deaf signers per sign (Caselli, Sevcikova Sehyr, Cohen-Goldberg, & Emmorey, 2017; Sevcikova Sehyr & Emmorey, 2019). For this database, signers rated how often they felt a sign appears in everyday conversation on a scale of 1 (very infrequently) to 7 (very frequently). The sign videos for the present study were selected from this database. For spoken language, familiarity ratings are highly correlated with corpora-based frequency counts (Gilhooly & Logie, 1980) and are consistent across different groups of participants (Balota, Pilotti, & Cortese, 2001). For sign languages, Fenlon, Schembri, Rentelis, Vinson, and Cormier (2014) found that subjective frequency ratings of British Sign Language (BSL) from Vinson, Cormier, Denmark, Schembri, and Vigliocco (2008) were positively correlated with objective frequency counts from the BSL Corpus, although the sample size for this analysis was much smaller than for spoken languages.

Parallel to spoken languages, faster lexical decision times are reported for signs that are rated as very frequent than for signs rated as infrequent (e.g., Carrieras et al., 2008; Caselli, 2015; Emmorey, 2002). Further, high-frequency signs are retrieved faster than low-frequency signs in picture-naming tasks (Baus & Costa, 2015; Emmorey, Petrich, & Gollan, 2012; Emmorey et al., 2013). Higher frequency signs are also acquired earlier by deaf children (Caselli & Pyers, 2017), and later acquired signs tend to be lower frequency (Vinson et al. 2008). In addition, high-frequency signs, like high-frequency words, tend to be shorter in duration (e.g., Börstell, Hörberg, & Östling, 2016) and are more likely to undergo coarticulation processes, such as sign lowering (e.g., Russell, Wilkinson, & Janzen, 2011). To date, similar linguistic and behavioral effects of lexical frequency have been found for signed and spoken languages.

In the present study, we utilized ERPs to investigate the impact of lexical frequency on sign comprehension. One limitation of using reaction times (RTs) to assess linguistic factors that affect lexical processing is that RTs reflect the final outcome of lexical access, including decision processes (e.g., Grainger & Jacobs, 1996). In contrast, ERPs continuously reflect information processing in real time, providing insight into the temporal neural dynamics of phonological (form) processing, lexical access, and sign comprehension. No study to our knowledge has examined how lexical frequency impacts the neural response in sign comprehension; however, Baus and Costa (2015) investigated frequency effects in a sign-production ERP study in which hearing bilinguals fluent in spoken Spanish or Catalan and Catalan Sign Language (LSC) named pictures in either Spanish/Catalan or LSC. The authors reported that high-frequency signs elicited more negative amplitudes than low-frequency signs in a 280–350-ms time window over occipital sites. This pattern parallels the frequency effect for spoken word production, although the difference between high- and low-frequency words may emerge earlier for speech (e.g., Strijkers, Costa, & Thierry, 2009). In contrast, for visual and auditory word comprehension, low-frequency words tend to elicit more negative amplitudes than high-frequency words (e.g., Dufau, Grainger, Midgley, & Holcomb, 2015; Dufour, Brunellière, & Frauenfelder, 2013; Kutas & Federmeier, 2011; Winsler, Midgley, Grainger, & Holcomb, 2018). Winsler et al. (2018) conducted a large megastudy of spoken word recognition (~1,000 words; 50 participants) using both a lexical decision task and a semantic categorization task similar to the one used in the present study. For the semantic decision task (detect an occasional animal word), Winsler et al. (2018) reported greater ERP negativities for low-frequency words at frontal and central sites, beginning 500 ms after word onset, which persisted into the final analyzed epoch, 800–900 ms. Here, we investigated whether the effects of sign frequency are parallel to the effects of word frequency with respect to the polarity, scalp distribution, and timing of ERPs to visual-manual signs.

We note that auditory word recognition is more parallel to sign recognition than visual word recognition because both speech and sign unfold over time and written words are a secondary code derived from speech and acquired later through instruction. An early study by Kutas, Neville, and Holcomb (1987) compared ERP responses to semantic anomalies in written, auditory, and signed sentences and found a strong similarity in the N400 component across modalities (greater negativity for anomalous than expected lexical items; see also Capek et al., 2009), but there were also differences, with a more prolonged ERP response for both auditory words and signs compared to written words. Grosvald, Gutierrez, Hafer, and Corina (2012) found that pseudosigns elicited a larger sentence-final N400 response compared to semantically appropriate signs, while nonlinguistic grooming gestures (e.g., scratching one’s nose) elicited a large positivity. This result highlights the linguistic specificity of the N400 component for signs. Further, Meade, Lee, Midgley, Holcomb, and Emmorey (2018) reported both semantic and phonological priming effects in the N400 window for single signs. Together, these results indicate that the N400 elicited by signs is sensitive to both phonological structure and lexical semantics. Based on these findings, we predict that lexical frequency will modulate ERPs in the N400 window, with low-frequency signs eliciting greater negativity than high-frequency signs, as found for spoken languages.

In addition to lexical frequency, the parallel megastudy by Winsler et al. (2018) examined the effect of relative concreteness on ERPs during auditory word recognition. Concrete spoken words elicited larger negativities than abstract words, with robust effects emerging after 400 ms that were widely distributed around central sites. Greater negativity for concrete than abstract words within the N400 window has been interpreted as reflecting richer semantic representations for concrete words that arise from associations with imagistic and sensorimotor representations and from larger semantic networks (e.g., Holcomb, Kounios, Anderson, & West, 1999; Kutas & Federmeier, 2011). Behaviorally, concrete words are typically recognized faster than abstract words (e.g., Kroll & Merves, 1986), possibly due to their semantic richness. For sign language, Emmorey and Corina (1993) found that concrete ASL signs were recognized faster than abstract signs in a lexical decision task. No study to our knowledge has investigated the effect of concreteness on ERPs during sign recognition. However, given the behavioral concreteness effects found by Emmorey and Corina (1993) and the sensitivity of the N400 to semantic manipulations in sign language (Capek et al., 2009; Kutas et al., 1987; Meade et al., 2018; Neville et al., 1997), we anticipate that concreteness effects within the N400 window will pattern like spoken language, with greater negativity associated with more concrete signs.

In addition to lexical frequency and concreteness, we examined the effect of iconicity on ERPs to signs. Iconicity values were obtained from deaf signers who rated the iconicity of the signs in the ASL-LEX database (Caselli et al., 2017; Sevcikova Sehyr & Emmorey, 2019). The number of deaf participants rating each sign varied between 26 and 31. Parallel to the subjective frequency ratings, participants were asked to rate each sign video on a 7-point scale based on how much the sign looks like what it means (1 = *not iconic at all,* 7 = *very iconic*). Several behavioral studies have used this type of rating to investigate the effect of iconicity on sign comprehension and production. In picture-naming tasks, several studies have now found that highly iconic signs are retrieved faster than noniconic signs (Baus & Costa, 2015; McGarry, Mott, Midgley, Holcomb, & Emmorey, 2018; Navarrete, Peressotti, Lerose, & Miozzo, 2017; Vinson, Thompson, Skinner, & Vigliocco, 2015). However, for comprehension the effects of iconicity have been mixed. Bosworth and Emmorey (2010) found that iconic signs were not recognized more quickly than noniconic signs in a lexical decision task. In a translation task, Baus, Carreiras, and Emmorey (2013) found that for proficient signers, iconic signs were actually recognized more slowly than noniconic signs. In a picture-sign matching task, Thompson, Vinson, and Vigliocco (2009) and Vinson et al. (2015) reported faster decision times when the iconic properties of the sign were aligned with visual features in the picture (e.g., the ASL sign BIRD depicts a bird’s beak and matches a picture of a bird with a prominent beak) compared to nonaligned pictures (e.g., a bird in flight where the beak is not visible). Thompson, Vinson, and Vigliocco (2010) found that form decisions about handshape (straight or curved fingers) were slower for more iconic signs, while Vinson et al. (2015) found that decisions about movement direction (up or down) were faster for more iconic signs. To date, the data suggest that iconicity does not have a clear, consistent impact on sign recognition.

To our knowledge, the only ERP study to explicitly manipulate iconicity in a sign comprehension task with deaf signers is Mott, Midgley, Holcomb, and Emmorey (2020). Mott et al. used ERPs and a translation priming paradigm (English word prime–ASL sign target) to investigate the effects of iconicity on sign recognition in proficient deaf signers and hearing L2 learners. Participants decided whether word-sign pairs were translation equivalents or not. For hearing learners, iconic signs elicited earlier and more robust priming effects (i.e., greater negativities for target signs preceded by unrelated word primes than by translation primes) compared to noniconic signs. In contrast, for deaf signers, iconicity did not modulate translation priming effects either in RTs or in the ERPs within the N400 window. The fact that priming effects did not begin earlier for iconic than noniconic signs suggests that iconicity does not facilitate lexical access for deaf signers, in contrast to L2 learners. Here we explore whether iconicity modulates ERPs to signs in a comprehension paradigm that does not involve priming or a translation task.

In sum, the purpose of the present study was to use ERPs to investigate how lexical frequency, concreteness, and iconicity affect the temporal neural dynamics of sign recognition. Following the “megastudies” of auditory and visual word recognition by Winsler et al. (2018) and Dufau et al. (2015), we gathered data from a large number of items and participants and treated these lexical variables as continuous measures, rather than categorizing and factorially manipulating them. This method avoids potential experimenter bias in selecting cut-off boundaries when categorizing continuous variables and allows for statistical analyses that control for the effects of other variables, such that results can clearly be attributed to the variable of interest (see Balota, Yap, Hutchison, & Cortese, 2012, for a discussion of the advantages of this type of “megastudy”). Following Winsler et al. (2018) and Emmorey, Midgley, Kohen, Sevcikova Sehyr, and Holcomb (2017), we used linear mixed-effects regression (LMER) techniques, rather than more traditional analyses, which allowed us to use single trial EEG data, rather than averaged ERP data. We also used linear mixed-effects (LME) models to visualize the ERP effects by computing an LME equivalent to scalp voltage maps using the *t* statistics at each electrode (see Data Analysis).

## MATERIALS AND METHODS

### Participants

Forty deaf ASL signers participated in this study (22 females; mean age = 28.9 years; *SD* = 7.2 years; range = 19–46 years). Thirty-one participants were native signers who were born into a deaf signing family, eight participants had hearing parents and were exposed to ASL before three years of age, and one participant learned ASL at age 12 years. Four participants were left-handed. Most participants were from San Diego or Riverside, California, and were compensated $15 per hour of participation. An additional eight participants were run but were not included in the analyses due to high-artifact rejection rates, very noisy EEG data, or failure to perform the task. Informed consent was obtained from all participants in accordance with the institutional review board at San Diego State University.

### Materials

The critical stimuli were 404 ASL sign videos from the ASL-LEX database (Caselli et al., 2017). An additional 26 probe sign videos (also from ASL-LEX) that referred to people were also presented (e.g., MAN, NURSE, MOTHER). Each sign occurred twice for a total of 52 probe signs. The critical stimuli varied in lexical class: nouns = 50%, verbs = 25%, adjectives = 16%, adverbs = 2%, and other (“minor” closed class signs) = 7%. All stimuli can be viewed on the ASL-LEX website (http://asl-lex.org). The Entry IDs (English glosses) for the signs are provided in the Supporting Information.

For frequency measures, we used the subjective frequency ratings from ASL-LEX, which used a scale of 1 (*very infrequent*) to 7 (*very frequent*). For the sample of critical signs, frequency ratings ranged from 1.63 to 6.84, with a mean of 4.50 (*SD* = 1.07).

Because no database with concreteness ratings is available for ASL signs, we used ratings from Brysbaert, Warriner, and Kuperman (2014) based on the English translations of the ASL signs. However, there were 13 signs that did not have translation equivalents in Brysbaert et al. (e.g., STARBUCKS, MCDONALDS, EUROPE), and therefore we gathered additional concreteness ratings for these words from 39 students at San Diego State University, using the same 5-point scale as Brysbaert et al. and mixing these 13 words in with 37 other words that varied in concreteness. Concreteness ratings for the English translation equivalents of the ASL signs ranged from 1.22 to 5.0, with a mean of 3.42 (*SD* = 1.60).

Finally, iconicity ratings were collected from deaf ASL signers (Sevcikova Sehyr & Emmorey, 2019) on a scale of 1 (*not iconic*) to 7 (*very iconic*), as part of ASL-LEX 2.0 (the ratings will be publicly available on the website in 2020). Iconicity ratings ranged from 1.0 to 7.0, with a mean of 3.03 (*SD* = 1.60).

The mean length of the videos was 1,770 ms (SD = 260 ms; range = 934–2,903 ms). The mean onset of the sign was 497 ms after the start of the video (SD = 122 ms; range = 200–1,168 ms). Sign onset is typically defined as when the hand(s) makes contact with the target location on the body (see Caselli et al., 2017, for details on how sign onset is determined). Sign offset is typically defined as the last video frame when the hand contacts the body before moving back to a resting position (see Caselli et al., 2017). The mean sign length was 506 ms (*SD* = 161 ms; range = 134–1,301 ms). We note that sign onset and length were related at least in part to this particular model’s sign production (rather than inherent to the signs themselves). Thus, they were not analyzed as experimental variables. However, given the variability of timing in the videos, sign length and sign onset were used as covariates in all analyses to control for some of the possible differences in EEG signal due to timing differences in the videos.

### Procedure

**Figure 1.**
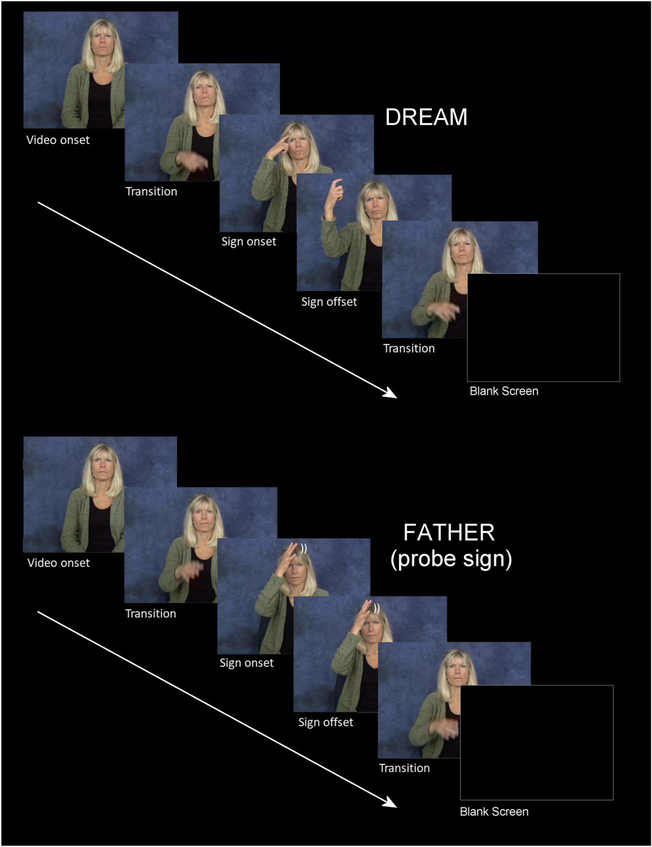
A schematic for two typical trials. Top is a critical sign trial and bottom is a “probe” trial (people sign) requiring a button press. Note that in this figure the images are still frames extracted from actual sign videos shown to participants.

Participants were seated in a comfortable chair, 150 cm from a 24-inch LCD stimulus monitor in a sound-attenuating darkened room while engaging in a go/no-go semantic categorization task. The testing session began with a short practice block of 15 trials, followed by two experimental blocks for 259 trials each. On each trial an ASL sign was presented as a video clip that was centered on the LCD monitor. Trials were of varying duration depending on the length of the individual video clips. Regardless of clip duration, a fixed blank-screen inter-stimulus-interval of 620 ms was interspersed between the offset of one clip and the onset of the next (see Figure 1 for a schematic of the paradigm). Each experimental block contained 202 critical target signs and 26 randomly intermixed probe signs (so-called people signs [e.g., BOY, NURSE]—12% of trials). Participants were instructed to press a button resting in their lap whenever they detected a people sign (i.e., a “go” stimulus) and to passively view all other “no-go” signs. On average every 12 trials a visual “blink” stimulus was presented for 2.5 s. This indicated that the participant could blink/rest their eyes, thus reducing the tendency for participants to blink during the critical sign ERP epochs.

### EEG Recording

**Figure 2.**
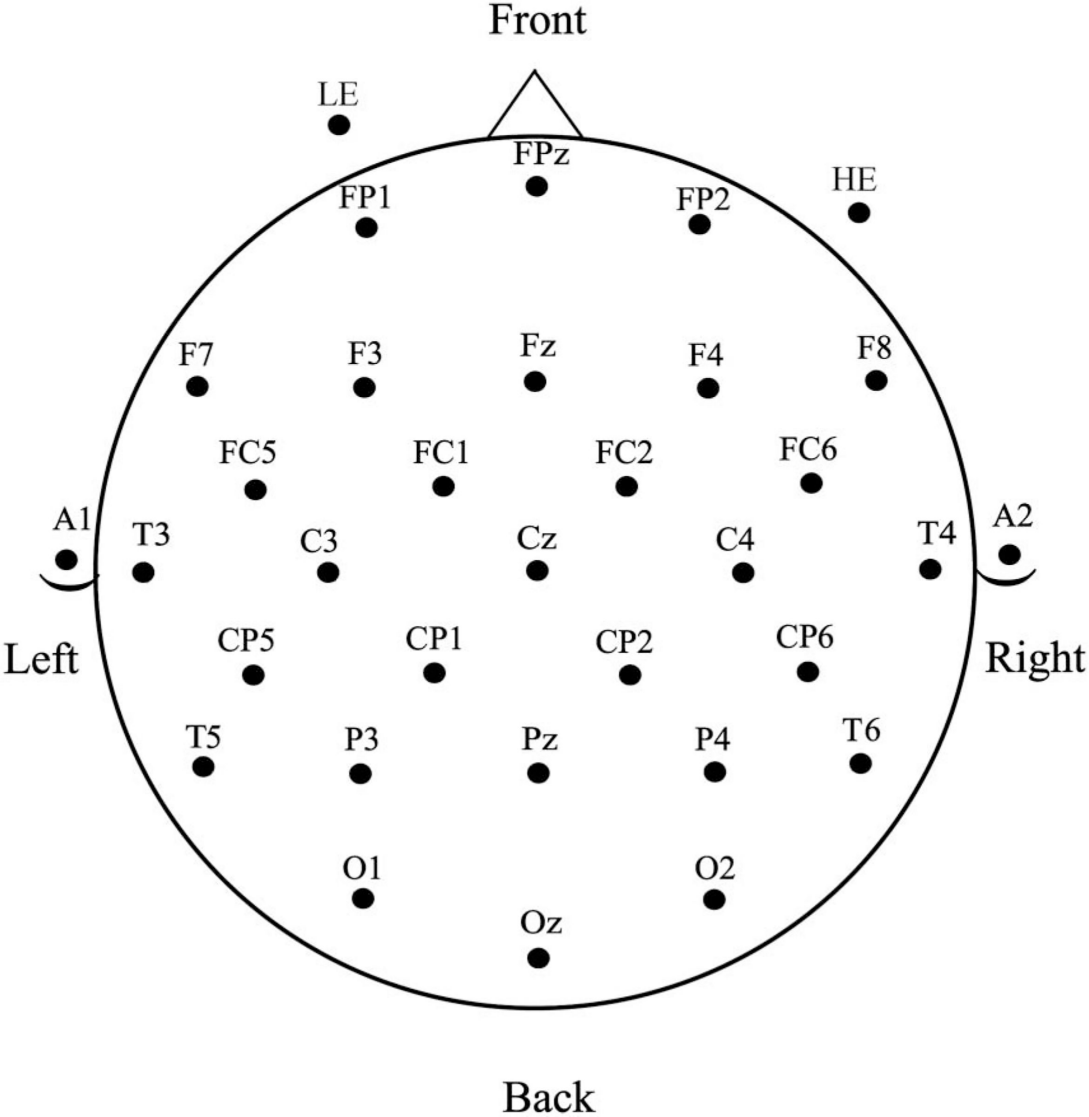
Electrode montage used for EEG recordings.

The EEG was collected using a 29-channel electrode cap containing tin electrodes (Electro-Cap International, Inc., Eaton, OH), arranged in the International 10–20 system (see Figure 2). Electrodes were also placed next to the right eye to monitor horizontal eye movements (HE) and below the left eye (LE) to monitor vertical eye movements and blinks. Finally, two electrodes were placed behind the ears over the mastoid bones. The left mastoid site was used as an online reference for the other electrodes, and the right mastoid site was used to evaluate differential mastoid activity. Impedances were kept below 2.5 kΩ for all scalp and mastoid electrode sites and below 5 kΩ for the two eye channels. The EEG signal was amplified by SynAmpsRT amplifier (Neuroscan-Compumedics, Charlotte, NC) with a bandpass of DC to 200 Hz and was continuously sampled at 500 Hz. Prior to data analysis the raw EEG data were corrected for blink and horizontal eye artifact using ICA (EEGLAB, Jung et al., 2000). Single-trial ERPs were formed from artifact-free trials, starting 100 ms prior to the onset of each ASL sign video and continuing for 1,200 ms. The 100 ms pre-sign-onset period was used as a baseline.

### Data Analysis

The data were analyzed using LMER, a relatively new approach to analyzing EEG data. LMER modeling is particularly advantageous for designs such as the current one, where there are multiple, potentially collinear, continuous variables. The correlation matrix for the variables included in our analysis is given in the Supporting Information (Appendix A1). Further, LMER allows for the model to simultaneously include random effects for both participant and item (see Baayen, Davidson, & Bates, 2008; Barr, Levy, Scheepers, & Tily, 2013). The models described below were fit using the lme4 package (Bates, Mächler, Bolker, & Walker, 2015) in R (R Core Team, 2014), and were structured based on models of Winsler et al. (2018).

EEG data were measured per participant, per item, and per electrode as average voltage over 100-ms epochs, starting in a 100–200-ms epoch, and continuing through an 1,100–1,200-ms epoch. Identical models were fit to predict mean amplitude for each of the 11 time windows. These models contained main effects for the three experimental variables, Frequency, Concreteness, and Iconicity, as well as for the two covariates for Sign Length and Sign Onset. Each of these five variables were standardized prior to analysis. Interactions between experimental variables were not included in the model. Although it is likely that these variables interact in ways that are detectable in the EEG signal, a full analysis of interactions would greatly increase the complexity of the models and is outside the scope of the present article. The questions of interest here relate to probing the broad patterns of effects related to sign-level variables. Fewer exploratory experiments will be necessary to adequately answer questions about how these variables interact with each other.

To analyze the distribution of the effects in addition to their overall effects, all electrodes were included in the models separately, each with three distributional variables corresponding to the spatial location of the electrode. These dimensions (X-position, Y-position, and Z-position) were included as interaction terms with each of the experimental variables and the covariates, as well as included as covariates themselves. Thus, the models had four parameters for each variable, an overall effect across all electrode sites, one for how the effect differs from left to right (X-position), one for how the effect differs between anterior and posterior sites (Y-position), and one for how the effect differs across electrodes lower on the scalp (e.g., T3, Oz) versus higher, central sites (e.g., Cz). See the Supporting Information (Appendix A2) for model code. This was the strategy used by Winsler et al. (2018) and was shown to appropriately analyze broad patterns of ERP distributions. Given the exploratory nature of the present experiment and the low spatial resolution of EEG signals, this approach was adopted to identify the general pattern of effects, without strong a priori hypotheses. But it should be noted that this strategy has limited power to detect especially focal or nonlinear interactions between effects and their distributions over the scalp.

The random-effect structure included random intercepts for participant, item, and electrode. Additionally, there were by-participant random slopes for the effect of each experimental variable (Frequency, Concreteness, and Iconicity), as well as Sign Length and Sign Onset.

To assess significance of each effect, confidence intervals were generated for each parameter. Additionally, *p* values were obtained for each parameter using type-two Wald tests, which allowed us to test the partial effect (unique variance) of each variable of interest. These *p* values were FDR (false discovery rate) corrected using the Mass Univariate Analysis Toolbox (Groppe, Urbach & Kutas, 2011). Effects were only interpreted as significant if they were significant by confidence interval (interval not containing 0) and by FDR-corrected *p* value (*p* < 0.05).

### Data visualization

**Figure 3.**
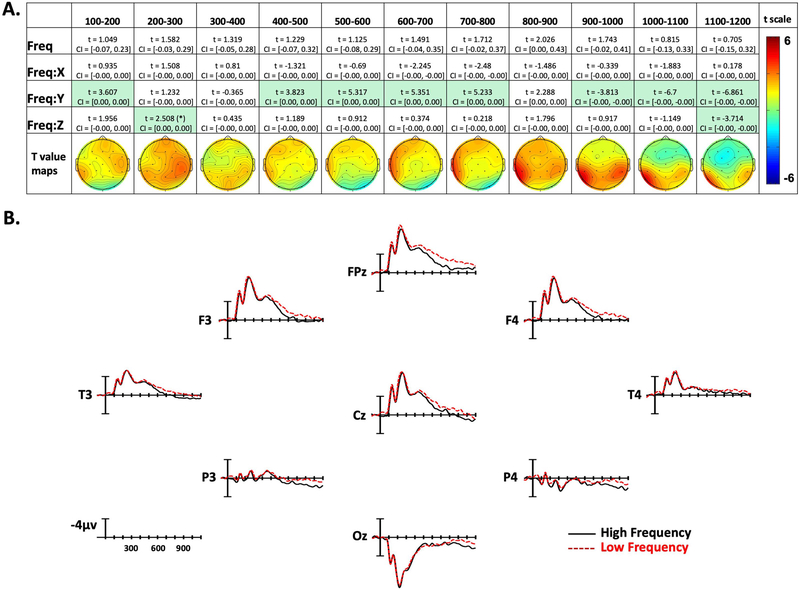
American Sign Language frequency effects. (A) linear mixed-effect *t* statistics, confidence intervals, and topographical *t-*statistic maps for frequency effects. Effects are only highlighted if results were significant with both confidence intervals and false discovery rate corrected *p* values; trend (*p* < .06) indicated by (*). (B) Frequency ERP plots made using the top and bottom quartiles of items sorted by frequency.

**Figure 4.**
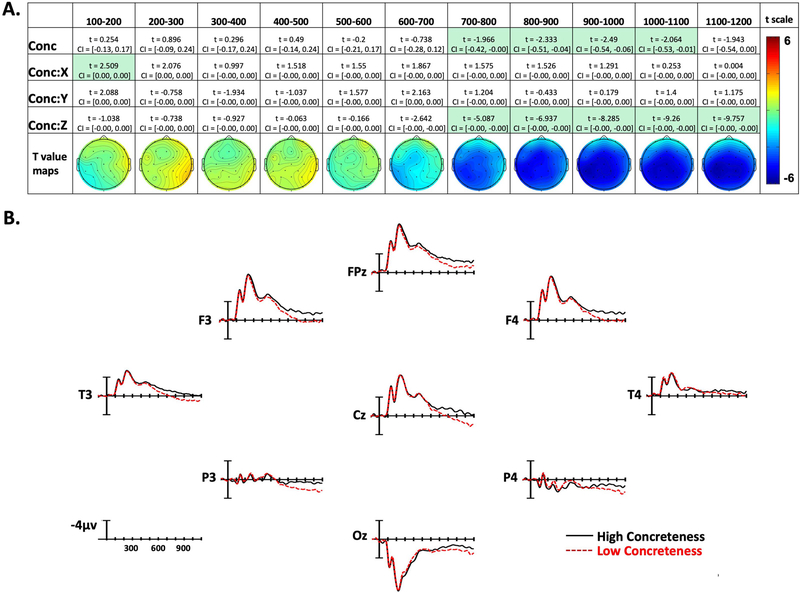
Concreteness effects. (A) Linear mixed-effect *t* statistics, confidence intervals, and topographical *t-*statistic maps for concreteness effects. Effects are only highlighted if results were significant with both confidence intervals and false discovery rate corrected *p* values. (B) Concreteness ERP plots made using the top and bottom quartiles of items sorted by frequency.

**Figure 5.**
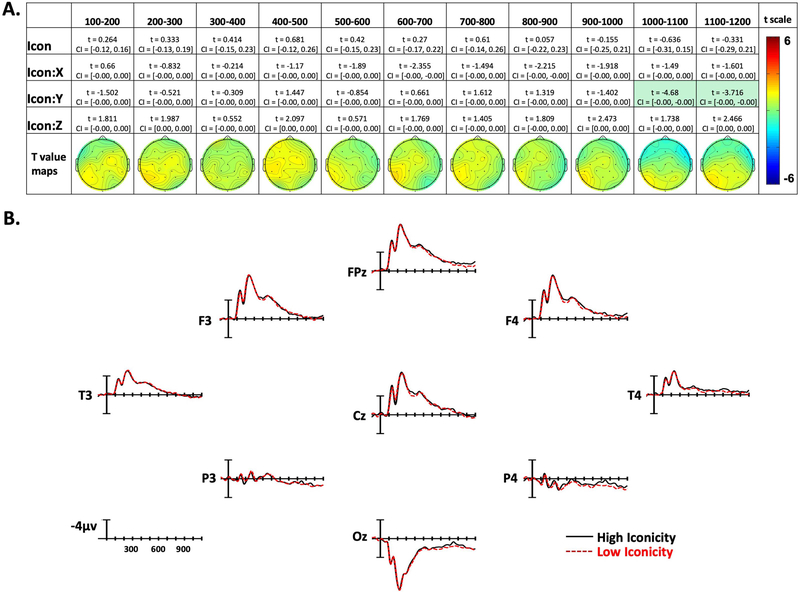
Iconicity effects. (A) Linear mixed-effect *t* statistics, confidence intervals, and topographical *t*-statistic maps for iconicity effects. Effects are only highlighted if results were significant with both confidence intervals and false discovery rate corrected *p* values. (B) Iconicity ERP plots made using the top and bottom quartiles of items sorted by frequency.

The confidence interval and *t* statistic for each parameter of interest are presented for each time window in Figures 3A–5A. The effect is highlighted if it was significant with both the confidence interval and the FDR-corrected *p* value. To visualize the distribution of the effects, models were constructed for each time epoch and electrode separately, and *t* values for the effect of each variable of interest were plotted across the scalp as topographic maps (maps in Figures 3A–5A). These models included the overall effects of Frequency, Concreteness, and Iconicity, as well as Sign Length and Sign Onset as covariates. The electrode-specific models also contained random intercepts for participants and items. Additionally, for visualization, traditional ERPs were plotted by averaging EEG data from 50 representative signs for the high and low conditions of each of the three experimental variables (see Figures 3B–5B). These averages controlled for the other experimental variables such that each comparison differed significantly only by the variable of interest, but not by the other two experimental variables. Note that these ERPs are for visual reference only and have not been analyzed statistically.

## RESULTS

### Behavioral Results

Participants correctly detected an average of 87% of the probe signs that referred to people, with an average false alarm rate of 4%. The mean RT for detecting the probe signs was 1,392 ms (*SD* = 82 ms).

### Linear Mixed-Effect Regression Results

Confidence intervals and *t* statistics for each parameter estimate are presented in a table for each variable of interest (Frequency, Concreteness, and Iconicity) in Figures 3A–5A. Table cells are highlighted if the effect is statistically significant both by its confidence interval, and FDR-corrected *p* value. To aid the visualization of the effects, for each time point there is a topographical map made from *t* values obtained from per-electrode LMER models. Additionally, Figures 3B–5B present averaged ERPs comparing each variable with 50 items per average, balancing for the other two variables.

### Frequency Effects

In the first epoch from 100–200 ms, there was a Frequency by Y-position interaction. As shown in Figure 3A, this indicates that lower frequency signs tended to produce more negativity in frontal sites, and less negativity in posterior sites. In the following two time windows, there were no significant effects. For the next four epochs, between 400 and 800 ms, there was again an interaction with the Y dimension, with low-frequency signs generating greater negativity in anterior electrode sites (see Figure 3A). In the following 800–900-ms epoch there were no significant effects. Based on the topographic maps in Figure 3A, the frequency effect seems to be transitioning from the previous anterior distribution, to a more posterior distribution in the following epochs. In the 900–1,000-ms time window there was again a Frequency by Y-position interaction, but now in the opposite direction as previously (note the flipped *t* statistic), indicating lower frequency items elicited more negativity in posterior sites. This effect remained through the final epoch (1,100–1,200 ms). Additionally, in the final epoch there was a Frequency by Z-position interaction, showing more negativity to low-frequency signs in peripheral sites, and a slight positivity to low-frequency signs in central sites.

### Concreteness Effects

In the 100–200-ms epoch there was a Concreteness by X-position interaction, with more concrete signs producing greater negativity on the left side of the montage, and less negativity on the right side. However, for the next four epochs (200–600 ms), there were no significant effects of concreteness. Beginning in the 600–800-ms epoch and continuing through the final epoch (1,100–1,200 ms), there was a Concreteness by Z-position interaction. This interaction indicates that concrete items elicited more negativity than abstract items, and this effect was distributed in the center of the scalp (see Figure 4A and Figure 4B). Additionally, in the four time windows between 700 and 1,100 ms, there was an overall effect of concreteness, showing that in these epochs the concreteness effect is distributed across the entire scalp.

### Iconicity Effects

In the first nine time windows analyzed, there were no effects of Iconicity. In the final two epochs (1,000–1,100 ms and 1,100–1,200 ms) there were Iconicity by Y-position interactions showing greater negativity to low-iconicity signs in posterior sites, and less negativity in anterior sites (see Figure 5A and Figure 5B).

## DISCUSSION

This is the first ERP study to investigate the effects of lexical frequency, concreteness, and iconicity on the temporal neural dynamics of sign comprehension. LMER models were fit in 100 ms-time epochs with per-participant, per-trial, per-electrode data to analyze the electrophysiological effects of these lexical variables on sign recognition. The results revealed both universal properties of lexical processing that are shared across signed and spoken languages, as well as different patterns that may be attributable to characteristics of the auditory-oral and visual-manual modalities. As predicted, lexical frequency and concreteness exhibited similar electrophysiological effects for sign recognition as previously found for spoken word recognition, but the time-course and scalp distribution of these effects were somewhat different for signs. No significant effects of iconicity were found, except for a weak effect in the late epochs.

### Frequency

ERPs were time-locked to video onset, and sign onset occurred approximately 500 ms later. Therefore, the very early effects of frequency observed in the first epoch (100–200 ms and 200–300 ms) are most likely associated with the transitional movement of the signer’s hand(s) from the resting position on her lap to the target location of the sign (see Figure 1). In these early epochs, lower frequency signs produced greater negativities than higher frequency signs at frontal and central sites. Unlike spoken languages, the linguistic articulators for sign languages are fully visible, and psycholinguistic research has shown that signers are sensitive to early linguistic cues that are visible in the transitional movement from a resting position to sign onset, as well as in the transitional movement between signs. For example, in gating studies signers can often identify the handshape and location of the sign prior to the onset of the sign, both when signs are presented in isolation (Emmorey & Corina, 1990; Grosjean, 1981) and when presented in a sentence context (Clark & Grosjean, 1982). Further, in an ERP study of sentence processing in German Sign Language, Hosemann, Herrmann, Steinbach, Bornkessel-Schlesewsky, and Schlesewsky (2013) found that the onset of the N400 response to sentence-final anomalous signs occurred prior to sign onset and thus had to be elicited by information present during the transition phase. We suggest that the very early effect of lexical frequency observed during the transition phase for isolated signs in the present study may reflect sensitivity to the frequency of sublexical properties, particularly handshape. Caselli et al. (2017) reported that handshape frequency was positively correlated with lexical frequency (i.e., higher frequency handshapes occurred in more frequent signs), but location frequency was not correlated with lexical frequency. If signers recognize a sign’s handshape during the transition phase, then it is possible that less frequent handshapes elicit a more negative response compared to more frequent handshapes (that occur in more frequent signs).

Frequency effects next emerged in the 400–500-ms epoch at frontal sites (slightly left lateralized), and then there was a later, more central-posterior frequency effect that began to emerge in the 800–900-ms epoch. We suggest that the different timing and distribution of these two effects may reflect sensitivity to frequency at two distinct levels: phonological form and lexical-semantics. For spoken languages, frequency effects are known to occur at multiple levels, including phonological encoding and lexical-semantic processing (e.g., Knobel, Finkbeiner, & Caramazza, 2008; Winsler et al., 2018). Previous ERP studies investigating implicit and explicit phonological priming in ASL indicate a frontal distribution for form priming, with smaller negativities over anterior sites for sign pairs that overlap in form (e.g., share the same handshape and location) compared to unrelated sign pairs (Meade, Midgley, Sevcikova Sehyr, Holcomb, & Emmorey, 2017; Meade et al., 2018). These results lead us to hypothesize that this earlier anteriorly distributed effect is related to accessing the phonological form of signs.

The later central-posterior distribution is more typical of the frequency effect observed in the N400 window for spoken language, which is usually associated with lexical-semantic processes. Note that this later effect is significant in the 900–1,000-ms epoch, which is 400 ms after the average sign onset (i.e., 500 ms after stimulus onset). It may be possible to observe separate effects of phonological frequency and lexical-semantic frequency in the ERPs to signs because phonological form encoding involves recognition of large movements of the hands and arms and distinct body configurations. The neural regions involved in form processing may be more neurally segregated from regions involved in lexical-semantic processing for sign language compared to spoken language. For spoken language, temporal cortex is involved in both phonological and lexical-semantic processing (e.g., Hickok & Poeppel, 2007), whereas for sign language more distinct neural regions appear to be involved in phonological processing (parietal cortex) and lexical-semantic processing (temporal cortex; see MacSweeney & Emmorey, 2020). In addition, the timing of these processes may be more segregated for sign language because the articulators are visible during the transition to sign onset. For speech, word onset coincides with stimulus onset, whereas there is ~500-ms delay between stimulus (video) onset and sign onset that contains form information about the upcoming sign. Future work that separately manipulates phonological and semantic variables will help to determine whether the distinct timing and distribution of the frequency effects observed here are linked to different processing levels involved in sign recognition.

### Concreteness

A robust effect of concreteness began to emerge 700–800 ms after video onset (~200 ms after sign onset) and continued throughout all analyzed epochs. The polarity of the effect (more negative for more concrete signs) and the wide distribution around central electrode sites parallel what has been found for spoken languages (e.g., Holcomb et al., 1999; Winsler et al., 2018). ERP effects of concreteness on word recognition are typically interpreted as reflecting richer semantic representations and denser links to associated semantic representations for more concrete words compared to more abstract words (Holcomb et al., 1999; West & Holcomb, 2000). Larger negativities to concrete words may result from increased neural activation arising from the more extensive semantic networks of these words, although greater N400 negativity does not appear to be monotonically associated with an increasing number of semantic features (Amsel & Cree, 2013; Kounios et al., 2009). Abstract words presented in isolation (as in the current study) may receive less semantic processing because they activate a smaller number of associations that may not be easily integrated into a unified concept (Barsalou & Wiemer-Hastings, 2005). In addition, semantic processing of concrete words engages a larger number of neural networks that are linked to sensorimotor properties of the concept (e.g., Barber, Otten, Kousta, & Vigliocco, 2013; Binder, Desai, Graves, & Conant, 2009). The parallel ERP results for signs and words indicate that language modality does not impact the neural networks that underlie processing of concrete vs. abstract concepts.

The time course of the concreteness effect likely reflects how the perception of single signs (produced in isolation) unfolds over time. A robust effect of concreteness emerges in the 700–800-ms time window (see Figure 4A), which is ~200 ms after the average sign onset (i.e., when the hand reaches the target location on the face/body or in neutral space). We suggest that some signs have already been recognized at this time window (Emmorey & Corina, 1990), giving rise to the concreteness effect. There is a main effect of concreteness for the next four epochs, and we suggest that this timing is consistent with the N400 concreteness effect observed for spoken and written word recognition.

### Iconicity

There were no significant effects of iconicity on ERPs until the final two epochs (1,000–1,200 ms). In these late epochs, the effect of iconicity was relatively weak (compared to the effects of frequency and concreteness) and consisted of a more negative response for less iconic signs at posterior sites. This finding is consistent with the results of Mott et al. (2020) who reported a late effect of iconicity when deaf signers performed a word-sign translation task. Specifically, noniconic signs exhibited a weaker translation priming effect (i.e., larger negativity for signs preceded by unrelated than by related English primes) compared to iconic signs in time windows that followed lexical by access (i.e., after the N400 window where translation priming was observed, but there was no interaction with iconicity). Following Mott et al., we suggest that the weak, late effect of iconicity reflects postlexical sensitivity to sign iconicity, perhaps reflecting a strategic effect when making the semantic categorization judgment. The (weak) iconicity effect emerged about 200–300 ms prior to the average RT for the “go” probe decision.

Our results indicate that the degree of form-meaning mapping does not impact the temporal neural dynamics of sign recognition and lexical access. In contrast to lexical frequency and concreteness, there does not appear to be a neural response that is modulated by lexical variation in iconicity during sign comprehension. Although sign iconicity may impact performance on tasks such as picture naming (e.g., Navarrete et al., 2017) or picture-sign matching (e.g., Thompson et al., 2009), iconicity does not appear to have a general impact on the neural networks that support sign recognition (see also Bosworth & Emmorey, 2010).

For spoken language, Lockwood, Hagoort, and Dingemanse (2016) found that iconicity (sound symbolism) impacted ERPs for new learners, specifically Dutch speakers who learned Japanese ideophones (marked words that depict sensory imagery; Dingemanse, 2012) in either a “real” condition (the correct Dutch translation) or in an “opposite” condition (the Dutch translation had the opposite meaning of the ideophone). Ideophones (auditorily presented) in the real condition elicited a larger P3 component and late positive complex compared to ideophones in the opposite condition. Further, these effects were greater for individuals who were more sensitive to sound symbolism (as assessed in a separate task). For native Japanese speakers, Lockwood and Tuomainen (2015) compared ERPs to iconic adverbs (adverbial ideophones) and arbitrary adverbs while participants made sensibility judgments to visually presented sentences that differed only in the type of adverb. Iconic adverbs elicited a greater P2 response than arbitrary adverbs, and there was a long-lasting late effect of iconicity, which the authors interpreted as a late positive complex. The authors speculated that the P2 effect arises from the integration of sound and sensory information associated with the distinctive phonology of ideophones and the later effect may reflect facilitated lexical access for arbitrary adverbs compared to ideophones.

However, ideophones differ from iconic signs because ideophones occur in sparse phonological neighborhoods (due to their distinctive phonology; Dingemanse, 2012), whereas iconic signs tend to be found in dense phonological neighborhoods (Caselli et al., 2017) and are not phonologically marked. In addition, highly iconic ASL signs tend to be found in dense semantic neighborhoods, whereas highly iconic English words are associated with sparser semantic neighborhoods (Thompson, Perlman, Lupyan, Sevcikova Sehyr, & Emmorey, 2020). Thus, the effect of iconicity on ERPs does not appear to be parallel for signed and spoken languages. However, no study that we know of has investigated whether continuous lexical variation in iconicity as measured by iconicity ratings of spoken words (e.g., Perry, Perlman, & Lupyan, 2015) modulates ERP components associated with spoken or written word recognition.

### The Temporal Neural Dynamics of Sign Recognition: Neurobiological Effects on Lexical Access

Our results revealed neurobiological principles that hold for both signed and spoken languages, as well as neural patterns that are modulated by language modality. The early waveforms shown in Figures 3B–5B (100–300 ms postvideo onset) reveal that signs elicit an occipital P1 response followed by an N1 response—both components are typically elicited by visual stimuli, including written words (Luck, 2014). Within these two early epochs, we observed effects of sign frequency (Figure 3A), which we attributed to signers’ sensitivity to the frequency of handshapes that are perceived during early transitional movements. This interpretation is consistent with the results of a MEG study by Almeida, Poeppel, and Corina (2016) in which deaf signers and hearing nonsigners were asked to discriminate between still images of possible signs and anatomically impossible signs. The earliest visual cortical responses (M100 and M130) were sensitive to this distinction only for deaf signers who also outperformed the nonsigners on the discrimination task. The authors concluded that extensive sign language experience (and/or deafness) can “shape early neuronal mechanisms that underlie the analysis of visual communication, likely on the basis of highly articulated, predictive internal models of gesture and language processing” (p. 372).

As can be seen in Figures 3B–5B, the N1 component was followed by the N300, a component that has been observed in studies using pictures or gestures (rather than written or spoken words) and is hypothesized to be involved in processing early visual semantic features (e.g., Hamm, Johnson, & Kirk, 2002; McPherson & Holcomb, 1999; Wu & Coulson, 2007, 2011). As found for picture and gesture processing, the N300 to signs has an anterior distribution. Meade et al. (2018) found both phonological and semantic priming effects on the N300 (reduced negativities for target signs preceded by related versus unrelated prime signs). Here, we observed frequency effects emerging during this component (400–500 ms postvideo onset), and we interpreted this early anterior effect as reflecting form-based lexical frequency, that is, accessing visual-manual phonological representations. It is possible that the N300, like the N250 for visual words, indexes the mapping between sublexical and lexical representations. Further research is needed to determine the functional significance of the N300 component for sign recognition and the factors that modulate this response.

As found for spoken word recognition, the N400 response to signs tends to be prolonged, compared to the N400 elicited by visually presented words. This is likely due to the fact that both spoken words and signs are dynamic and unfold over time. During these later epochs (~400 ms postsign onset), the anterior frequency effect shifted to a more widely distributed posterior effect that we interpreted as reflecting lexical-semantic frequency. Concreteness effects were also observed during these later epochs with the same polarity (i.e., more negative for more concrete signs) and the same distribution as observed for spoken languages. Our findings support the consensus that the N400 component is associated with amodal lexical-semantic processing (Kutas & Federmeier, 2011). The results are also consistent with ERP studies demonstrating N400 effects for lexical-level semantic violations in signed sentences (Capek et al., 2009; Grosvald et al., 2012; Gutierrez, Williams, Grosvald, & Corina, 2012; Kutas et al., 1987; Neville et al., 1997).

Finally, lexical variation in iconicity did not modulate the neural response during sign recognition, suggesting that this lexical variable is not represented in the brain in a manner that is parallel to either frequency or concreteness. Despite the pervasiveness of iconicity in ASL (Thompson et al., 2020), there does not appear to be a general neural response that is associated with variation in iconicity. However, the present study was only designed to identify general patterns of effects and may not have been able to detect particularly focal effects or nonlinear interactions with iconicity. Thus, further work is necessary to determine under what conditions (if any) sign iconicity impacts lexical access and sign recognition and/or if there are particular types of iconicity that might modulate the neural response to signs, such as perceptual or motor iconicity (Perniss, Thompson, & Vigliocco, 2010) or highly transparent signs that are “manual cognates” with gestures (Ortega, Özyürek, & Peeters, 2019; Sevcikova Sehyr & Emmorey, 2019).

In sum, we used a large-scale, item-based analysis with LMER models which controlled for the colinearity of lexical variables, and this approach allowed us to identify ERP effects that were specific to the continuous variables of lexical frequency, concreteness, and iconicity. Despite the perceptual and motoric differences between signed and spoken languages, the overall results indicate that very similar electrophysiological processes underlie lexical access for signs and words. The findings provide a better understanding of the timing and distribution of these lexical effects on sign recognition such that future studies can analyze them more precisely. We expect that future studies will be able to uncover nuances in the temporal neural dynamics of sign recognition based on the broad pattern of lexical effects presented here.

## Supplementary Material

Entry_IDs_ASL_Signs

Appendix A

Appendix B
