# Supplementary material for: Neurophysiological Correlates of Frequency, Concreteness, and Iconicity in American Sign Language
Source: Neurobiol Lang (Camb). 2020 Jun 1;1(2):249–67. doi: 10.1162/nol_a_00012 (PMC7544239; doi:10.1162/nol_a_00012)
Supplement: Supplementary file 2 [file nol-1-2-249-s002.pdf]

Appendix A

Variables	*M* (*SD*)	Freq.	Conc.	Icon.	Onset	Length
Frequency	4.50 (1.07)		-.30	-.14	-.05	-.25
Concreteness	3.42 (1.60)			.25	.07	.17
Iconicity	3.03 (1.60)				.01	.08
Sign Onset	497 (122)					-.05
Sign Length	506 (161)					

Appendix B

```
#### Example Model 100-200ms ####

lmer_100_200 <-lmer(MeanAmp_100_200 ~ Freq + Conc + Icon    #main effects
                 + Onset + Length                           #covariates
                 + X_pos + Y_pos + Z_pos                    #distribution covariates
                 + Freq:X_pos                               #2-way interactions
                 + Freq:Y_pos
                 + Freq:Z_pos
                 + Conc:X_pos
                 + Conc:Y_pos
                 + Conc:Z_pos
                 + Icon:X_pos
                 + Icon:Y_pos
                 + Icon:Z_pos
                 + (1 | SUBJECTS)                           #random intercepts
                 + (1 | ITEM)
                 + (1 | ELECTRODE)
                 + (0 + Freq | SUBJECTS)                    #random slopes
                 + (0 + Conc | SUBJECTS)
                 + (0 + Icon | SUBJECTS)
                 + (0 + Onset | SUBJECTS)
                 + (0 + Length | SUBJECTS),
                 data = Data)
```

This model was fit on mean amplitude measurements for each of the 11 time windows. The fixed effects were main effects for the three experimental variables (Frequency, Concreteness, Iconicity) and 2-way interactions between each of these variables and the three spatial variables (X-position, Y-position, and Z-position). The random effects were random intercepts for subject, item, and electrode, and by-subject random slopes for each of the three experimental variables and the two covariates (sign onset and sign length). The random slopes did not include covariance between random effects. Confidence intervals were obtained using the confint function, and *p*-values were obtained using the Anova function (Type two Wald chi-square tests) in the CAR package. *P*-values were then FDR-corrected using MATLAB software (see methods). *T*-values and CIs for each fixed effect are included in Figures 3A-5A, and they are highlighted if they were significant with both the CI and the FDR-corrected *p*-value.
